# Catching the tide at the flood or being in the right place at the right time

**DOI:** 10.1038/s41430-025-01602-4

**Published:** 2025-03-30

**Authors:** D. Joe Millward

**Affiliations:** Department of Nutrition, Exercise, Chronobiology & Sleep, School of Biosciences & Medicine, Faculty of Health and Medical Sciences, University of Surrey, Guildford, UK

**Keywords:** Feeding behaviour, Nutrition


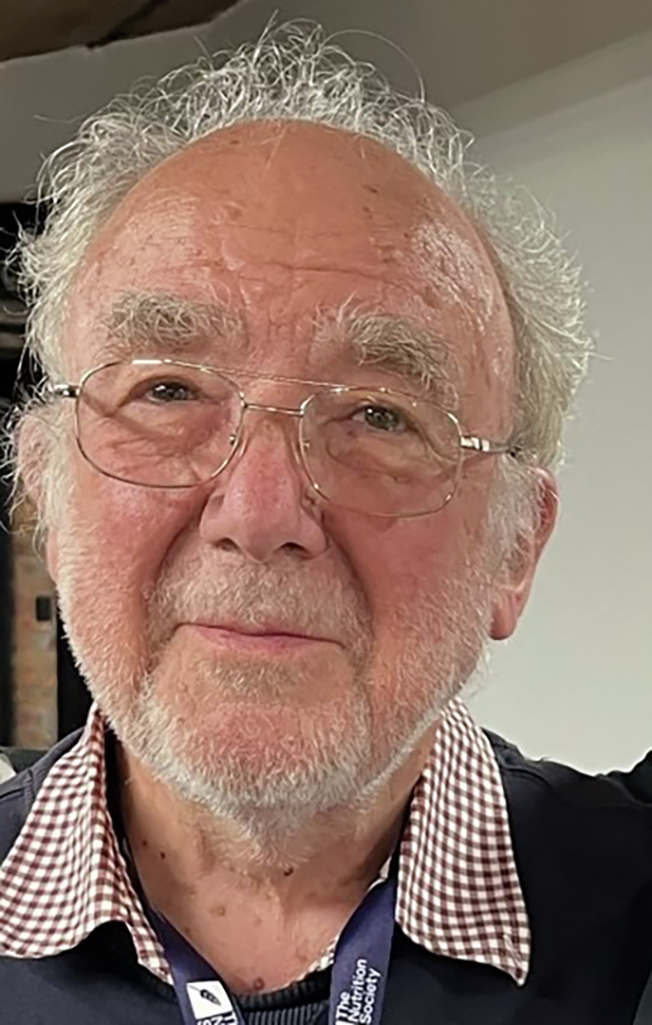


Brutus’s oft-quoted words to Cassius make sense when the tide is recognisable, and ambition is strong. However, often this is not the case and the tide is caught by accident, through being in the right place at the right time. So began my own professional journey. I had missed the tide several times during my early life leaving my Grammer School at 16 to work as a technician chemist for five years while attending day-release courses. The wake up call came during 6 months bed rest while recovering from pulmonary TB with recognition that alternative pathways were available. I chose a 4 year University course at Cardiff with a Biochemistry degree in 1966, during which time one event proved life changing. This was leading the Welsh contingent, one of five busloads of students, overland to India in the summer of 1965, culminating in all 200 of us in our sleeping bags in the Viceregal Lodge in Simla at the height of the India-Pakistan war over Kashmir. Prior to my finals the following year, I wrote to John Waterlow, head of the MRC Tropical Metabolism Research Unit in Jamaica about the possibility of a PhD studentship with him, recounting an interest in malnutrition observed whilst driving a bus to India. This clinched the studentship and my professional career began. Contrary to the view of my tutor at Cardiff that all I would be doing would be counting coconuts on the beach, the TMRU was a globally recognised hot house of nutrition and metabolism research with state of the art equipment, and a pioneering work ethos. One of my tasks on arrival was to align and recalibrate a 4πwhole-body liquid scintillation counter, unique in the world, to measure [^40^K] and whole body potassium in the infants [1]. This was so sensitive that when the Chinese tested their first hydrogen bomb in December, 1966 I registered an increase in the background radioactivity count within a few days. I also inherited a mass spectrometer for [^15^N] whole body protein turnover work from my predecessor Dave Halliday which I had to maintain.

I had caught the tide of global protein deficiency seen by the UN in 1968 as an Impending Crisis [2], and the current was protein turnover. Waterlow had shifted focus from fatty liver disease to protein malnutrition [3], sharing the standard view that Kwashiorkor, the often fatal oedematous malnutrition, was a consequence of protein deficiency which prevented the proper regulation of protein synthesis and turnover, impairing cellular function. Although as subsequently argued by Mike Golden, protein deficiency was inconsistent with the successful management of such infants with a low protein, maintenance energy regime fed with antibiotics, key electrolytes & micronutrients, which Ann Ashworth, David Picou, George Alleyne and John Garrow had developed, Waterlow maintained his view for the rest of his life. Waterlow and Joan Stephen developed a constant intravenous infusion of [U-^14^C] lysine which allowed accurate in vivo measurements of protein turnover in the whole body and tissues, [4], but the methodology was laborious and progress was slow. After introducing me to several amino acid labelling methods to study turnover in vivo, [^75^Se] labelled seleno-methionine and [6-^14^C] arginine, he left it up to me to develop a suitable method. I did this by using a single large dose of [^14^C]Na_2_CO_3_ to label the carboxyl groups of aspartate and glutamate in proteins and measure their decay rate over subsequent days. The intense metabolism of these labelled groups after their liberation following proteolysis dramatically reduced the risk of recycling of label so that the decay rates of these two amino acids in labelled proteins reflected the true turnover rate. This work [5, 6], considered at the time to be biochemically elegant made me famous and at a 1969 Wellcome Trust meeting at the TMRU in 1969 I met Vernon Young and showed him my results. He incorporated them into a review he was writing [7] and we became lifelong friends. However the method proved much less practical than the precursor-product approach and I never used it again.

After a 1 year post doc in the Biochemistry department at UCL, I moved to the London School of Hygiene, (LSHTM) in 1970 with John Waterlow after his appointment to the departmental Chair on the death of Ben Platt in 1969. There Waterlow established through the Wellcome Trust, the Clinical Nutrition and Metabolism Unit (CNMU) at the Hospital for Tropical Diseases at St Pancras in London which was formally opened in 1973 by Princess Alice, Countess of Athlone, (aged 90), who, in 1955 as Chancellor of the University of West Indies had opened the TMRU in Jamaica. This new unit provided some continuity for Waterlow’s work and soon became another internationally-recognised centre of excellence. As recently described [8], working initially under Waterlow with Peter Garlick and then establishing my own group we adopted the precursor-product approach to study protein turnover in vivo, initially with tail-vein tracer infusions of U-^14^C tyrosine, and then with a single flooding dose of [^3^H] phenylalanine. This allowed rates of tissue protein synthesis to be measured accurately, and allowed turnover to be calculated from comparison of the synthesis rate with the rate of changes in tissue protein content in response to interventions, with a step change in the range and volume of studies in animal models. By the time of my move to Surrey to take the Chair in Human Nutrition in 1992, we had a complete description of protein turnover in murine muscle during development, in response to dietary and hormonal manipulations and infection, and in response to stretch-induced skeletal muscle hypertrophy in an adult avian model as well as an account of the extent of muscle-bone interactions in response to dietary protein and energy deficiency [9]. In parallel with this animal work, starting in the late 1970s, we applied stable isotope tracer kinetic approaches with [^13^C-1] leucine, [^15^N]glycine, [^2^H_5_]phenylalanine and [^2^H_2_]tyrosine to human studies of protein synthesis in muscle, to whole body protein homoeostasis in response to varying protein intakes and to post prandial protein utilisation(PPU), initially in collaboration with Mike Rennie and Dave Halliday and then with Paul Pacy. This work allowed the first formulation of the protein stat model for growth regulation in 1995 [10], which has recently been updated [8, 9, 11]. The studies of PPU were especially important for the development of the adaptive metabolic demand model for protein requirements during maintenance [12]. At Surrey my research diversified up to my full retirement in 2009 after which I maintained consultancy work for Public Health England writing much of the 2011 Energy Requirements Report [13] and continued to teach, as a visiting Professor of Medical Nutrition, at St Georges University Medical School, Grenada till 2017 and at the LSHTM, UCL and at Surrey, up to the present time.

A consequence of developing methodologies to study protein turnover and of the analytical and critical ethos of the TMRU and the LSHTM, was concern that inappropriate methods can be misleading and that data can be incorrectly interpreted so that what becomes established concepts are based on very shaky foundations. For example it was proposed that the urinary excretion of 3-methyl histidine, (3MH), a post translationally modified amino acid present mainly in actin which is not reutilised for protein synthesis could be used to measure muscle protein breakdown, as the 3MH/creatinine ratio [14]. This lead to global adoption of this method. However we showed that in the rat the majority of urinary 3MH derived from the rapidly turning over smooth muscle and other non muscle actin myofilament pools [15], and that in boys with Duchenne Muscular Dystrophy with an increased urinary 3MH/creatinine ratio consistent with the common assumption that muscle proteolysis was increased, we showed with [^13^C-1] leucine infusion that muscle protein synthesis and turnover was markedly depressed, [16]. In fact by the time the 1989 Rank Prize was awarded to Young & Munro for their work on 3MH, the method was no longer used.

The nutritional issue which has been my main focus in terms of lack of rigour in its development has been protein and amino acid requirements and protein quality evaluation. Shortly after my arrival at the LSHTM, the 1971 FAO/WHO expert consultation on Energy and Protein requirements was convened, [17], with both Philip Payne and John Waterlow from the LSHTM as members. Waterlow chaired the FAO/WHO/UNU 1981 consultation [18], on which the UK 1991 DRVs for protein and energy were based and to which I contributed [19], and I co-chaired with Peter Garlick, the 2002 consultation on protein and amino acid requirements, writing some but not all of it [20]. I also participated in the 1989 [21] and 2011 [22] FAO/WHO protein quality consultations, (the reports from these various meetings were published in 1973,1985 and 2007 and 1991 and 2013 for the protein quality reports and these dates will be used here for the subsequent discussion). Thus these various consultations have been the backdrop to my professional life for more than 40 years. As I wrote in 1997 [23] “Few issues in nutritional science have aroused such long standing and deep-seated controversies as protein and amino acid requirements”. The 1973 report used a factorial method to identify the adult protein EAR as obligatory N losses +30% (i.e. 0.44 g/kg/d) with a safe intake as EAR + 30%, (0.57 g/kg/d), for adult men. This was lower than previous reports and rapidly became extremely contentious, especially at MIT [24], setting the agenda for the next 1985 report which increased the safe intake to 0.75 g/kg/d for adult men and women on the basis of a small number of “representative” short-term N-balance studies, and some long term (2-3month) balances. The report omitted the combined results of all short-term N-balance studies in the sub-maintenance to maintenance range reported from MIT, (131 balances in 107 men with a total of 7 intakes from 0.04-0.51gprotein/kg/d) [25] which in fact confirmed the 1973 EAR and safe intake see [26, 28]. The current adult protein requirement, deriving from the 2007report, (EAR = 0.66 g/kg/d, safe intake, 0.83 g/kg/d), is 10% higher than the 1985 values and derives from a meta-analysis of N-balance data [27]. Whilst this was comprehensive in terms of not excluding any published data, its analytical approach, linear regression of balance versus intake of individual subjects at 3 or more intakes, precluded consideration of the overall findings of Youngs 1973 paper [25], but as I have discussed elsewhere [28], included values some of which were biologically not sensible, (slopes negative or >1 or with positive intercepts). As a result, the overall range of individual requirement values were from −29 to +451 mgN/kg/d and a relatively reasonable safe intake could only be calculated from the median EAR value by excluding all variance except true between-individual variation derived statistically. The 1973 report noted that the safe protein intakes were the physiological requirements and likely to be lower than intakes observed in populations at energy balance consuming otherwise healthy diets, and provided an indication of how far intakes could fall before a significant risk of deficiency was likely to occur. This was correct for both the 1973 and the1985 recommendations but became more complicated with the 2007 report because of the issue of recommended amino acid requirements and protein quality.

The key issue was the adult indispensable amino acid (IAA) requirement pattern. In the 1973 report a marked fall with age in amino acid requirements were noted, with the adult values derived by Hegsted from his reanalysis of nitrogen balance studies, These were adopted again in the 1985 report which concluded that for adults only digestibility and not the amino acid score could influence dietary protein quality so that the main rationale for animal source proteins, (ASF), in the adult diet, their higher protein quality, disappeared. This precipitated another crisis and Vernon Young embarked on a programme of [^13^C] IAA balance studies starting with valine, leucine, lysine and threonine published in 1986 all suggesting much higher requirements, Shortly after John Rivers and I published an analysis of these and other studies [29]. As well as identifying major shortcomings in the [^13^C] IAA balance calculations, we compared the 1973/1985 adult IAA values with the obligatory oxidative losses (OOL) of IAAs, i.e. the theoretical pattern of amino acids mobilised from tissue protein to provide for the IAA metabolic demand in adults fed a protein-free diet, giving rise to the obligatory nitrogen loss (ONL). We argued that there was no reason why the pattern for the maintenance requirements should match that of tissue protein but if the IAA values were accurate, the OOL values would be similar to the IAA requirement value for the rate limiting IAA but would be higher for all other IAAs according to their relative excess in tissue protein compared with the requirement pattern. Thus values for the OOL for leucine and lysine, abundant in tissue protein, were 2 and 2.5 times higher than their requirement values, with the OOL for methionine + cysteine very similar to their requirement values: i.e. the ONL was driven by the demand for S-Aas. This was consistent with data showing that the addition of the S-AAs to a protein free diet reduced the ONL [29, 30]. John Waterlow, acting as an EJCN editor, had sent our paper to Vernon Young and at a London meeting where both of us were attending he got out his copy and pointed to the table of OOL values showing the much higher lysine value compared with all other IAAs, commenting that the adult lysine requirement value must be underestimated. Shortly after he published the MIT IAA requirement pattern [31] stating that the dietary intakes required to balance the OOL of IAAs, could be an initial guide to their minimum physiological requirement in healthy adults “a suggestion made by Millward and Rivers”, which was not what we had said [32]. While this debate was going on, in 1989 FAO convened a consultation to evaluate protein quality by the digestibility corrected amino acid score (PDCAAS) method [21] which I attended in Washington. The controversy over the MIT scoring pattern was noted and its use was not endorsed but neither was the 1973/1985 adult pattern: instead a scoring pattern for preschool children based on minimal data was adopted as an interim pattern. After this there was further stable isotope work on adult IAA requirements: Young eventually linked up with Anura Kurpad in Bangalore to conduct 24 h [^13^C-1] leucine balance studies, Pencharz & Ball in Toronto introduced the indicator amino acid oxidation (IAAO) method, and my own work focussed on protein homoeostasis and PPU identified above, and this became the backdrop for the 2007 WHO report on protein and amino acid requirements [20], where most of the work was critically evaluated (although not critically enough-see below). The 2007 report became the backdrop for the 2013 report on protein quality evaluation, for which I had summarised the main issues which were unresolved and made practical suggestions for a way forward [28, 33] which were unfortunately not heeded and yet another unsatisfactory report was issued [22]. Most recently I have revisited my earlier critique of the IAAO method with a much more detailed analysis [34]. This identified several fatal flaws in their design not previously identified, which in my view should exclude all IAAO-derived values for IAA requirements from any scoring patterns for human protein quality evaluation and negates any usefulness of IAAO-derived protein requirement values. This leaves us with the N-balance values and the 24 h [^13^C-1] leucine balance studies, appropriately conducted and interpreted [28, 33], as the only useful information about protein and IAA requirements.

## The future

As others in this series have commented, questions about food supplies, especially animal source proteins are a major issue both within the global climate crisis and because in 2023 one out of 11 people in the world, and one out of every five in Africa, faced hunger and over 3.1 billion people could not afford a healthy diet [35]. Even though the 1973/1985 recommendations for IAAs effectively removed the importance of protein quality for adults consistent with long term studies with wheat [36, 37] and soya [38] which showed maintenance of bodyweight and body composition, the meat and dairy industries maintain their powerful lobbying for the superiority of ASFs over plant source proteins, now quoting the illogical proposal of the 2013 protein quality report [22] to use uncapped amino-acid scores to rank the quality of individual proteins. With 75 to 80% of global soya production used for animal feed, the largest fraction of which comes from Brazilian rainforests, this is clearly unsustainable. The adaptability of human protein metabolism enabling demand to be adjusted to match intake and the presence of protein in most plant food sources means that consumption of ASFs is a matter of personal preference rather than nutritional necessity. Mike Gibney once commented to me that meat consumption will eventually be determined by its cost to the consumer but it is arguable that its cost to the planet is already too high.
